# Synthesis of Au@Pt Core—Shell Nanoparticles as Efficient Electrocatalyst for Methanol Electro-Oxidation

**DOI:** 10.3390/nano9111644

**Published:** 2019-11-19

**Authors:** América Higareda, Siva Kumar-Krishnan, Amado F. García-Ruiz, José Maya-Cornejo, José L. Lopez-Miranda, Daniel Bahena, Gerardo Rosas, Ramiro Pérez, Rodrigo Esparza

**Affiliations:** 1 Posgrado en Ciencia e Ingeniería de Materiales, Centro de Física Aplicada y Tecnología Avanzada, Universidad Nacional Autónoma de México, Boulevard Juriquilla 3001, Santiago de Querétaro 76230, Mexico; ame_libert.16@hotmail.com; 2 Cátedras CONACYT_Instituto de Física, Benemérita Universidad Autónoma de Puebla, Apdo. Postal J-48, Puebla 72570, Mexico; mksivakumar84@gmail.com; 3 UPIICSA-COFAA, Instituto Politécnico Nacional, Te 950, Col. Granjas-México, Iztacalco, Ciudad de México 08400, Mexico; amado.garcia@gmail.com; 4 Centro de Física Aplicada y Tecnología Avanzada, Universidad Nacional Autónoma de México, Boulevard Juriquilla 3001, Santiago de Querétaro 76230, Mexico; iqm_jamc@yahoo.com.mx (J.M.-C.); lopezfim@gmail.com (J.L.L.-M.); 5 Laboratorio Avanzado de Nanoscopía Electrónica (LANE), Centro de Investigación y de Estudios Avanzados del I.P.N., Av. Instituto Politécnico Nacional 2508 Col. San Pedro Zacatenco, Ciudad de México 07360, Mexico; dbahenau@cinvestav.mx; 6 Instituto de Investigaciones Metalúrgicas, UMSNH, edificio U, Ciudad Universitaria, Morelia Michoacán 58060, Mexico; grtrejo@umich.mx; 7 Instituto de Ciencias Físicas, Universidad Nacional Autónoma de México (UNAM), Av. Universidad s/n, Cuernavaca, Morelos 62210, Mexico; ramirop21@gmail.com

**Keywords:** gold-platinum nanoparticles, core–shell structure, solution-phase synthesis, electrocatalysis, methanol oxidation

## Abstract

Bimetallic Au@Pt nanoparticles (NPs) with Pt monolayer shell are of much interest for applications in heterogeneous catalysts because of enhanced catalytic activity and very low Pt-utilization. However, precisely controlled synthesis with uniform Pt-monolayers and stability on the AuNPs seeds remain elusive. Herein, we report the controlled deposition of Pt-monolayer onto uniform AuNPs seeds to obtain Au@Pt core–shell NPs and their Pt-coverage dependent electrocatalytic activity for methanol electro-oxidation. The atomic ratio between Au/Pt was effectively tuned by varying the precursor solution ratio in the reaction solution. The morphology and atomic structure of the Au@Pt NPs were analyzed by high-resolution scanning transmission electron microcopy (HR-STEM) and X-ray diffraction (XRD) techniques. The results demonstrated that the Au@Pt core–shell NPs with Pt-shell thickness (atomic ratio 1:2) exhibit higher electrocatalytic activity for methanol electro-oxidation reaction, whereas higher and lower Pt ratios showed less overall catalytic performance. Such higher catalytic performance of Au@Pt NPs (1:2) can be attributed to the weakened CO binding on the Pt/monolayers surface. Our present synthesis strategy and optimization of the catalytic activity of Au@Pt core–shell NPs catalysts provide promising approach to rationally design highly active catalysts with less Pt-usage for high performance electrocatalysts for applications in fuel cells.

## 1. Introduction

Direct methanol oxidation has great potential for next generation energy systems for applications in power conversion for the future use in the portable electronic devices [1]. Exploitation of highly efficient electrocatalysts for the sluggish anodic methanol oxidation reaction (MOR) is found to be primary research area for direct methanol fuel cells (DMFC) development. Platinum (Pt) has been commonly used as electrocatalyst, which play indispensable role in MOR catalysts owing to their tunable and selective electrocatalytic activities arising from their distinct electronic structure [2,3,4]. However, the availability of this critical material and its high-cost have greatly limited their use in large scale comercial applications and sustainability [5]. In addition, the Pt-based catalysts are easily poisoned by the generation of intermediates of carbon monoxide (CO) during the electrooxidation reaction of methanol, which propitiates complete cuts down the electrocatalytic activity and stability of the catalysts [6]. Substantial investigation has been directed toward development of new catalysts via alloying of Pt with other less expensive metals such as Pd, Ag, Au, Cu, etc., either as bimetallic alloys or core–shell architecture in order to reduce the Pt-utlization and, at the same time, to enhance significantly the electrocatalytic activity [7,8,9]. One promising strategy is to design a catalyst comprising of Pt-monolayer onto a less expensive metallic substrates (Pd, Ag, Au, Ir, Co, etc.) as core–shell structure, which enables 100% utilization of enriched Pt atoms onto the surface of the substrate and high cataytic efficiencies through substrate induced surface strain effects [7,10].

Among different substrate NPs, Pd NPs have been commonly employed for controlled deposition Pt-shells owing to its similar lattice constant (mismatch of 0.77%) and low-cost [11,12]. However, the stability of these Pd@Pt core–shell nanostructure based catalysts is found to be a key issue under long-term operational conditions resulting in leaching of core metal [13] and undergo undesirable surface segregation of Pt [14], which poses significant impact on their electrocatalytic performance and life-time of the electrocatalysts [15]. As such, there is a search for alternative substrate for deposition of Pt-monolayers for active and stable electrocatalysts. Recent studies have demonstrated that utilizing smaller AuNPs as a substrate for deposition of Pt offers an effective strategy which significantly suppress the electrocatalysts from degradation by up-shifting the dissolution potential of Pt, which in turn of exhibit long-term stability [16,17]. Notably, the integration of Pt with Au significantly forms a stable nanostructure as it exhibits strong electronic coupling between these two metal components. Suntivich et al. [18] demonstrated that the Au@Pt NPs possess tunable surface composition for MOR. Their study revealed that the Au_0.5_Pt_0.5_ exhibited higher electrocatalytic activity by controlling the surface atomic segregation of Pt atoms. Peng et al. [19] showed that the deposition of Pt-submonolayers on AuNPs and the Pt coverage-dependent electrocatalytic activity for MOR, also revealed that the low Pt coverage such as monoatomic-thick layers exhibited remarkably higher electrocatalytic performance and structural stability, whereas at higher Pt-shell thickness there was not any obvious increment found in the electrocatalytic activity. The surface Pt-rich bimetallic nanocatalysts with different Au/Pt atomic ratios have demonstrated enhanced hydrogenation selectivity or activity than AuPt alloys and monometallic Pt nanomaterials [20,21]. In addition, Au@Pt NPs showed the highest response current for the catalytic oxidation of glucose solution compared with those of Pt and Au NPs [22,23]. Recent theoretical predictions showed that the PtAu surface avoids the CO formation and apparently exhibits higher CO-poisoning tolerance when compared with the Pt@Pd surface [24].

The seed-mediated solution-phase approach has been commonly exploited for synthesis of wide range of core–shell nanoparticles [25,26]. Importantly, significant research efforts have been attempted to develop an Au@Pt core–shell nanostructures consisting of Pt-skin shells, and realizing their electrocatalytic performance for various catalytic reactions [16,18,19,27,28,29,30]. However, controlled growth of Pt shells onto substrate NPs still remains as a great challenge because of large lattice mismatch between the core and shell components and occurrence of galvanic replacement reaction between Pt precursor and metal substrates, which resulted in non-uniform deposition of Pt over AuNP substrates. Different synthetic approaches have been explored for the controlled deposition of Pt-monolayers on AuNPs such as surface limited redox replacement of an under potentially deposited (UPD) technique [31], electro deposition [32,33], epitaxial growth [10], and wet-chemical synthesis [29]. Besides these efforts, deposition of Pt submonolayers with well-controlled thickness and atomic segregation still remains a great challenge.

Herein, we report a facile, seed-mediated synthetic approach for controlled design of Au@Pt core–shell NPs using polyol technique. We varied the Au/Pt ratio in the Au@Pt core–shell NPs by changing the Pt precursor concentration in the synthesis and the effect of electrocatalytic properties for methanol oxidation was studied in alkaline media. The resultant Au@Pt NPs with ratio of 1:2 exhibited greatly enhanced electrocatalytic activity for methanol oxidation in comparison with the other as-synthesized Au@Pt core–shell NPs. This work not only demonstrates controlled synthesis of Au@Pt core–shell NPs and detailed STEM analysis, but also demonstrates the optimization of elecrocatalytic activity for methanol oxidation by precisely varying Au/Pt ratio.

## 2. Materials and Methods

### 2.1. Materials

Gold (III) chloride trihydrate (HAuCl_4_·3H_2_O), silver nitrate (AgNO_3_), chloroplatinic acid hexahydrate (H_2_PtCl_6_·6H_2_O), polyvinylpyrrolidone (PVP, MW: 40K), and ethylene glycol (EG). All chemicals were purchased from Sigma-Aldrich (Sigma-Aldrich, St. Louis, MO, USA) and were used as received without further purification. All the glassware containers were washed with acetone, Milli-Q water, and ethanol before use.

### 2.2. Synthesis of AuNP Seeds

The AuNP seeds were prepared using a well-known polyol method. First, we synthesized AgNPs seeds and these seeds were used to prepare AuNPs. In a typical synthesis, 2 mL of AgNO_3_ solution (20 mM) and 4 mL of PVP (50 mM) were added in 10 aliquots every two and half minutes under magnetic stirring into 10 mL of EG at 160 °C and maintaining the reaction for 1 h. Then, 0.5 mL of pre-synthesized AgNPs seeds were mixed with 5 mL of EG and heated to 160 °C. After reaching 160 °C, 0.5 mL of aqueous solution of HAuCl_4_ precursor (50 mM) and 1 mL of PVP (50 mM) were added in the same way as the previous procedure and the reaction continued for 1 h.

### 2.3. Synthesis of Au@Pt Bimetallic Core–Shell Nanoparticles

Au@Pt NPs were obtained using seed-mediated chemical reduction process. In a typical synthetic procedure for Au@Pt (1:1), 0.5 mL of Pt precursor (50 mM) and 1 mL of PVP (50 mM) were added in the same way as the previous procedure under magnetic stirring into the solution containing 7 mL of pre-synthesized AuNPs seeds at 160 °C, then the reaction temperature increased to 190 °C for 15 min. The resultant NPs samples were washed with ethanol and DI water by using centrifugation at 12,000× *g* rpm for 15 min. The final products were dispersed in isopropyl alcohol and used for the preparation of electrocatalysts and respective characterizations. Similar protocol was followed for synthesis of Au@Pt (1:2) and (1:3) except changing the concentration of Pt precursor from 0.5 mL to 1 and 1.5 mL, respectively.

### 2.4. Structural Characterization

X-ray diffraction (XRD) patterns were recorded on Rigaku Ultima IV diffractometer (Tokyo, Japan) with Cu Kα radiation (40 kV, 15 mA, 1.54051 Å); measurements were performed using parallel-beam geometry with 2θ scans from 30 to 80° at room temperature. The UV–vis spectra were recorded in a spectrophotometer Metash 6000 (Shanghai, P.R. China) in the wavelength range from 350 to 900 nm at room temperature.

The morphological and chemical characterization of the samples were accomplished using a Hitachi SU8230 cold-field emission scanning electron microscope (Hitachi High-Tech, Tokyo, Japan) equipped with energy-dispersive X-ray spectrometer XFlash^®^ 6/60 (EDS) Silicon Drift Detector system (Bruker, Billerica, MA, USA). EDS spectra were collected using the transmission mode of the electron microscope. Samples were deposited on Al stubs. On the other hand, structural characterization of the samples was carried out using JEM-ARM200F transmission and scanning transmission electron microscope TEM/STEM (Jeol, Tokyo, Japan), with a field-emission electron source and a CEOS spherical corrector (CEOS GmbH, Heidelberg, Germany) in the illumination system and accelerating voltage of 200 keV. Bright-field (BF)-STEM images and high-angle annular dark-field (HAADF)-STEM images were obtained using a camera length of 80 mm and a collection angle of 50–180 mrad was registered. The samples were prepared by dispersing a colloidal NPs solution and dropping a small amount of NP solution over carbon coated copper grids and dried well under ambient conditions.

### 2.5. Preparation of Electrocatalysts

Electrocatalysts were prepared by dispersing known amount of Au@Pt NPs solution with XC-72 Vulcan carbon by ultrasonication for 15 min, the amount of Au@Pt NPs was calculated to 20wt% of total metal phase. Then, the mixture was kept for magnetic stirring for 6 h at 60 °C. After supporting Au@Pt NPs samples with the XC-72 Vulcan carbon, the electrocatalysts were dried by freeze-drying.

### 2.6. Electrocatalytic Measurements

Electrochemical measurements were performed using a standard three-electrode electrochemical cell, using a BioLogic VSP potentiostat (Biologic, Seyssinet-Pariset, France) at room-temperature. A graphite rod and Hg/HgO electrode were used as counter electrode and reference electrode, respectively. The catalytic ink was obtained by mixing 1 mg of Au@Pt/C electrocatalyst, 70 µL of isopropyl alcohol and 7 µL of Nafion^®^ solution 117 (5 wt%) by ultrasonication for 15 min. To prepare the working electrode, 7 µL of catalytic ink was dropped onto the glassy carbon BASi electrode (diameter of 3 mm) and dried at room temperature. Cyclic voltammograms (CVs) were carried out at scan rate of 50 mV s^−1^ in alkaline medium (0.3 M KOH) and for methanol oxidation (1.0 M) at scan rate of 20 mV s^−1^. Before starting the electrochemical measurements, the solution was deaerated by bubbling nitrogen-purging for 10 min.

## 3. Results

### 3.1. Structural Characterization

**Figure 1:**
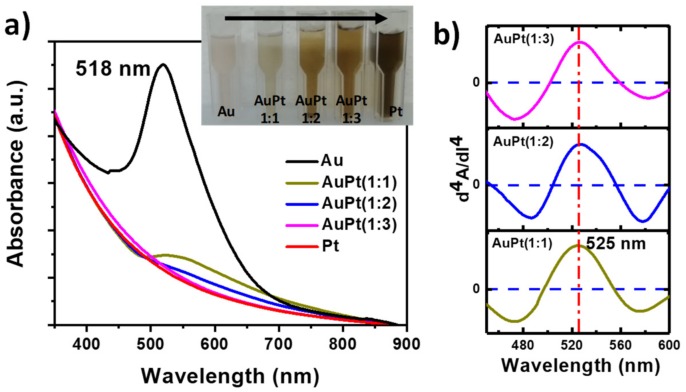
(**a**) UV–vis absorption spectra and (**b**) fourth-derivative absorption spectra of the obtained Au, Pt and Au@Pt NPs with different nominal compositions. The inset in Figure 1a shows a photograph of the corresponding samples.

The Au@Pt NPs with different ratio were prepared by a two-step synthesis process. In the first step, Au NPs seeds were obtained by the polyol method at 160 °C. In the next step, the pre-synthesized Au NPs were used a seed site to controlled deposition of Pt monolayers over Au NPs core. The optical response of the different pristine AuNPs and bimetallic NPs was studied by UV–vis spectroscopy analysis. Figure 1a display the UV–vis spectra of the Au, Pt and bimetallic Au@Pt core–shell NPs with different nominal compositions of (1:1), (1:2), and (1:3). The UV–vis spectrum of the pristine AuNPs exhibits a localized surface plasmon resonance (LSPR) peak at 518 nm, which corresponds to small AuNPs (less than 10 nm) [34]. After deposition of Pt-layers onto AuNPs, the major LSPR peak at 518 nm red-shifted to higher wavelength along with the significant decrease in the intensity (525 nm). As can see from the Figure 1a, Au@Pt (1:1) showed a smaller decrement in the LSPR peak, whereas Au@Pt of (1:2) and (1:3), the LSPR peaks were almost diminished. To further confirm the Pt-deposition, the color change in the result solution were monitored, which clearly indicated that the color of pristine AuNPs was progressively changed into dark brown color after Pt-deposition with different ratios, as displayed in photographic image in the inset of Figure 1a. Moreover, derivative UV-spectrophotometry is an analytical technique that uses first or higher derivatives of absorbance with respect to wavelength to obtain qualitative and quantitative analysis, mainly if there are unresolved bands or overlapping bands [35].

Figure 1b shows the fourth-order derivative spectra of the Au@Pt core–shell NPs with different nominal compositions (1:1, 1:2, and 1:3) where a positive band at maximum of 525 nm in each spectrum is observable. This band gives clear idea about the maximum wavelength of each spectrum. A characteristic of the fourth-order derivative is that has a sharper central peak than the original and that a weak and small absorbance bands can be identified. In the case of AuPt (1:3), where the absorbance band in the UV–vis spectrum is so weak, that it is not possible observed, however using the fourth-order derivate it is possible to appreciate the LSPR peak of the AuNPs. It is important to mention that to identify clearly the fourth-order derivative of the weak absorbance bands on the graphic; the intensity of each spectrum was normalized. These results confirm that the AuNPs exhibit covered Pt-layers with different shell thickness which shields the Au LSPR peak. This effect is reasonable because of the damping effect caused by Pt-shells, which is in line with the previous studies [36,37]. Furthermore, the structure and composition of the Au@Pt core–shell NPs were examined using parallel-beam X-ray diffraction (XRD) analysis.

**Figure 2:**
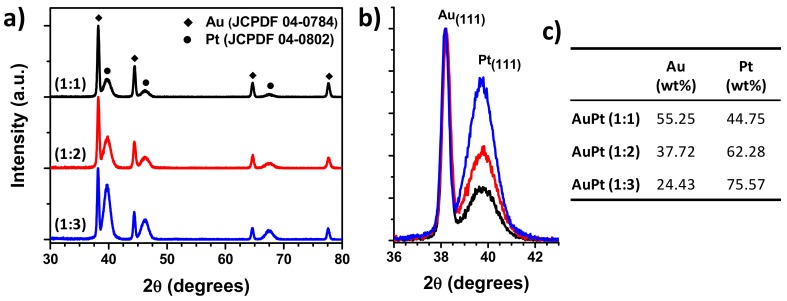
(**a**) XRD patterns of Au@Pt NPs with (1:1), (1:2), and (1:3) composition;(**b**) (111) reflections of Au and Pt structures; and (**c**) elemental composition of Au and Pt.

Figure 2a shows the XRD patterns of Au@Pt NPs maintaining constant the Au precursor and varying the molar ratio of Pt precursor to (1:1), (1:2), and (1:3). The XRD patterns showed that the reflection at 2θ values of 38.2°, 44.4°, 64.6°, and 77.6°, which can be indexed into (111), (200), (220), and (311) crystalline planes of the Au fcc structure (JCPDF 04-0784), respectively, and at 39.6°, 46.2°, and 67.5° which can be indexed into (111), (200), and (220) reflections of the Pt fcc structure (JCPDF 04-0802), respectively. Important to mention that bimetallic Au-Pt NPs with alloy structure, the XRD pattern shows reflections between the corresponding of Au and Pt, which are related to the composition [38,39]. In the present case, XRD diffraction patterns show reflections corresponding to position of monometallic Au and Pt, no peak position shifts are observed, lattice parameter *a* values are compared to their pure counterpart (0.4078 nm and 0.3926 nm for Au and Pt, respectively), and the intensity is dependent on the atomic ratio between them, confirming the Au@Pt NPs are mainly composed of crystalline phases of Au and Pt and suggesting the formation of core–shell structure. Figure 2b shows in detail of the (111) reflection of Au and Pt structures. Intensity and shape of the Pt reflections is lower and wider than the Au reflections, indicating the size of Pt crystallites is much smaller than that of Au crystallites, in addition, the intensity of Pt reflections increases from (1:1) to (1:3) composition, indicating that the thickness of Pt shell increased. Therefore, uniform core–shell structure of Au@Pt NPs with different thickness has been synthesized. This results are in accordance with the previous report by Lingyu Tan et al. [30], they reported that the synthesis of uniform and well-structured Au@Pt bimetallic nanoparticles with core–shell structure. Elemental composition of Au@Pt NPs was estimated using the Rietveld method with Profex program for BGMN [40]. The results are shown in the Figure 2c. The fact that the composition in the Au@Pt NPs may not follow the molar ratio exactly could be due to the differences in nucleation and growth rates for the individual metals.

**Figure 3:**
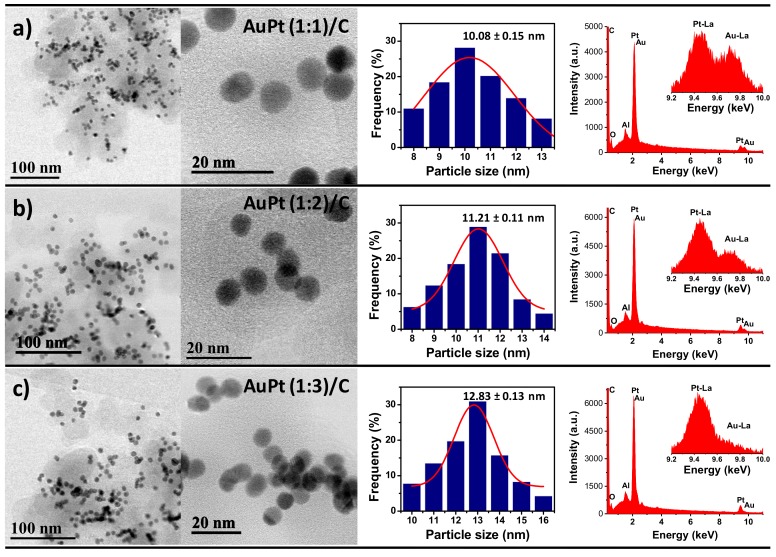
BF-STEM images, size distribution histograms and EDS spectrum of Au@Pt NPs supported on carbon black Vulcan XC-72R with different nominal atomic compositions: (**a**) (1:1), (**b**) (1:2), and (**c**) (1:3).

For electrochemical measurements, the Au@Pt samples were first loaded to carbon black Vulcan XC-72R. Figure 3 display the bright field scanning transmission electron microscopy (BF-STEM) images of the Au@Pt NPs of (1:1), (1:2), and (1:3) catalysts after supporting onto carbon Vulcan. It is clear that the Au@Pt NPs are homogeneously well-dispersed over the carbon support without any agglomeration. The as-synthesized AuNPs seeds showed uniform spherical shapes with an average particle size of 8.1 ± 0.11 nm (not showed). By varying the Au/Pt ratio (1:1, 1:2, and 1:3), the thickness of Pt monolayers can be effectively controlled, as shown in the Figure 3. Specifically, at optimized Pt-precursor concentration, the reduced Pt-atoms form Pt^2+^ can deposited onto AuNPs and followed by surface diffusion to other sites to generate Au@Pt core–shell NPs with ultrathin Pt-layers. Importantly, for Au@Pt (1:1), the presence of higher Au sites to deposition of low Pt ions, resulting formation of Au@Pt NPs with average particle size of 10.08 ± 0.15 nm (Figure 3a), which is greater than that of the original AuNPs seeds. The difference in the diameter is associated with the average thickness of the Pt-layers on the AuNPs, that corresponds approximately to three atomic layers of Pt-shells, which is in good agreement with previous report [41]. Energy-dispersive X-ray spectrum (EDS) indicates the presence of both elements with a nominal composition of Au/Pt 45.2/54.8wt%, which agrees with the obtained values from XRD analysis. More Pt ions were deposited onto fewer amounts of Au core sites leading to generation of Au@Pt core–shell NPs with higher Pt-shell thickness. Homogeneous particles were obtained for Au@Pt (1:2) (Figure 3b) and Au@Pt (1:3) (Figure 3c) with an average particle size distribution calculated of 11.21 ± 0.11 and 12.83 ± 0.13 nm, respectively, which is 3.11 and 4.73 nm greater in comparison with the AuNPs seeds. These differences in the diameter correspond approximately to five and eight atomic layers of Pt-shells for Au@Pt (1:2) and (1:3) NPs, respectively.

EDS spectra shows that the nominal composition when more Pt ions were deposited, the obtained values were Au/Pt 31.2/68.8wt% and Au/Pt 20.3/79.7wt% for Au@Pt (1:2) and (1:3) NPs, respectively, these values are close to the stoichiometric ratio of the precursors (1:2 and 1:3, respectively), reflecting the complete reduction of all the metal precursors. Previous studies demonstrated that the deposition of Pt onto AuNPs was dominated by the epitaxial growth mode (layer-by-layer growth) [41]. Specifically, the reduced Pt atoms are nucleated onto AuNPs surfaces and followed by the diffusion on the other surfaces of the nanoparticles. To accomplish the layer-by-layer deposition, the deposition rate should be lower than that of diffusion rate (V_dep_<V_diff_). Therefore, it is necessary to perform synthesis at relatively higher temperature (above 150 °C) in order to provide the sufficient energy for atoms to diffuse faster to the other faces of the substrate, leading to uniform conformal deposition of Pt onto the AuNPs seeds with uniform shell thickness [25]. Our present synthesis was performed at 160 °C and 190 °C to maintain the V_dep_<V_diff_ to obtain uniform and smooth deposition of Pt-shells. By adjusting the Pt-precursor amount, the Pt-shell thickness or ratio can be obtained.

**Figure 4:**
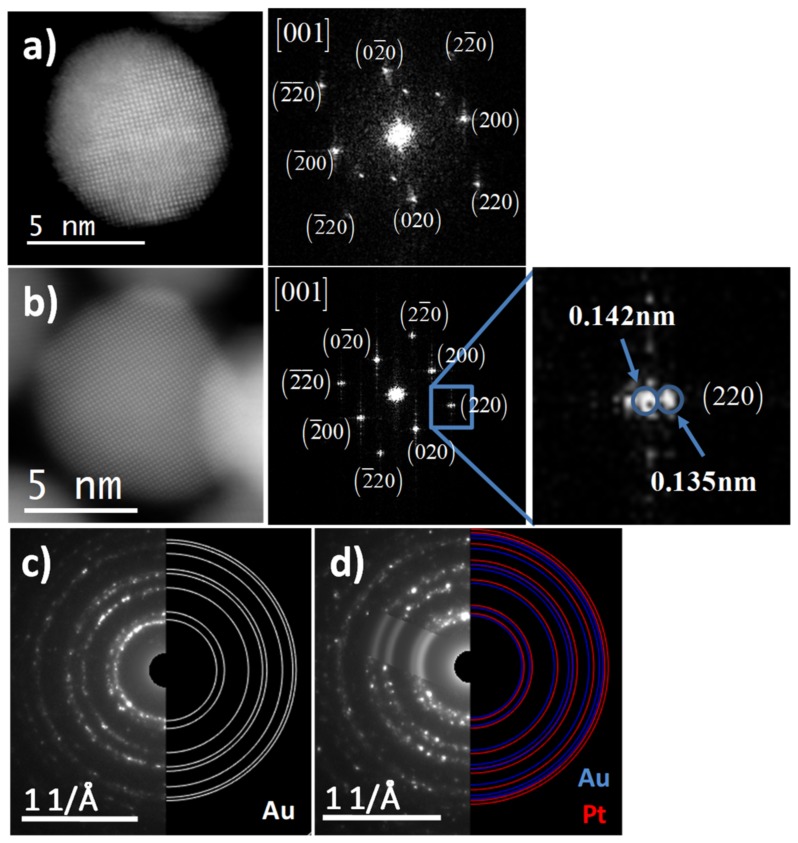
High-resolution HAADF-STEM images with their FFT of (**a**) AuNPs and(**b**) Au@Pt (1:3) NPs, and SAED patterns of (**c**) AuNPs and (**d**) AuPt (1:3) NPs.

Figure 4 shows high-resolution high-angle annular dark field scanning transmission electron microscopy (HAADF-STEM) images of AuNPs and Au@Pt NPs with different nominal atomic compositions. From the HAADF-STEM image of AuNPs (Figure 4a), the d-spacings 0.2032 and 0.1449 nm was obtained. Such d-spacings values correspond to (200) and (220) crystalline planes respectively, which show good agreement with the Au fcc structure (JCPDF 04-0784); fast Fourier transform (FFT) shows the main reflections confirming the [001] zone axis. The HAADF-STEM images revealed that the nanoparticles with spherical shape and smooth surface were retained after Pt-deposition, suggesting the epitaxial growth of Pt-monolayers. HAADF-STEM image of individual Au@Pt (1:3) particle in Figure 4b does not reveal a contrast difference between Au core and Pt shells. This is mainly because the Au and Pt have similar atomic number (79 and 78, respectively), therefore it is hard to distinguish Pt from Au. However, the measurements of the reflection spots of the FTT show the superposition of both Au and Pt structures. Inset of the FFT shows that the d-spacings 0.142 and 0.135 nm, which assigned to (220) plane of Au and Pt fcc structures, respectively.

Selected area electron diffraction (SAED) patterns were also obtained from the AuNPs and bimetallic AuPt (1:3) particles for phase analysis. Simulated diffraction rings with aligned diameters using the CrysTBox software were drawn on the experimental SAED patterns and the corresponding interplanar spacings were determined [42]. The simulated diffraction patterns excellently reproduce the experimental ones. The ring structure of SAED pattern of AuNPs (Figure 4c) indicated that the entire nanoparticles were nanocrystalline in nature and it is characteristic of polycrystalline gold [43]. The rings of SAED pattern were indexed according to (111), (200), (220), (311), (222), (400), and (331) reflections of Au fcc on the basis of their d-spacings of 0.2309 nm, 0.2026 nm, 0.1407 nm, 0.121 nm, 0.1162 nm, 0.1001 nm, and 0.0915 nm, respectively. These values of interplanar spacings were compared with the X-ray diffraction database (JCPDF 04-0784). Figure 4d shows the SAED pattern of the bimetallic AuPt (1:3) particles with the superposition of the simulated diffraction rings of Au and Pt structures. The diffraction rings of the SAED pattern are thicker and not well defined in comparison with the SAED pattern of the AuNPs, it due to that the lattice parameter being very close.

**Figure 5:**
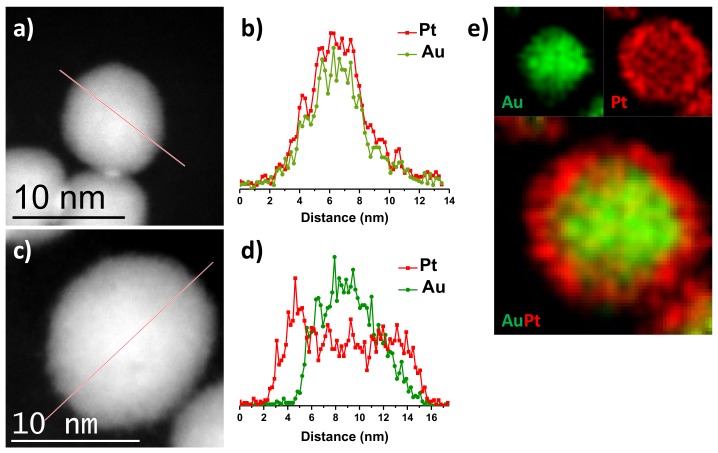
(**a**) HAADF-STEM image, (**b**) EDS line-scan profile of Au@Pt (1:1) NPs, (**c**) HAADF-STEM image, (**d**) EDS line-scan profile, and (**e**) EDS elemental mappings of Au@Pt (1:3) NPs. Green represents Au component and red represents Pt component.

The core–shell structure of Au@Pt NPs was verified by STEM-EDS. Figure 5a shows a HAADF-STEM image of Au@Pt (1:1) NPs and its corresponding line-scan plot (Figure 5b) to clarify that the signal of Au is confined to core region whereas the Pt signal is uniformly distributed throughout the entire particle, confirming the formation of a core–shell structure. These results are consistent with the previous reports, when few atomic layers are deposited on atomic core NPs with the atomic ratio Au/Pt is 1:1 [44,45]. Figure 5c shows a HAADF-STEM image of Au@Pt (1:3) NPs; as above was mentioned due to similar atomic number between Au and Pt, the HAADF-STEM image does not reveal a contrast difference between Au core and Pt shells. However, the EDS line-scan and mapping analyses were carried out by scanning along the NPs to identify the presence and position of both Au and Pt elements in the nanoparticles. When the concentration of Pt ions increased, the layers of Pt on the surface of Au core can clearly be observed in Figure 5d,e. The EDS line-scan profile (Figure 5d) evidences the core–shell structure of Au@Pt (1:3) nanoparticle; the Au trace has one peak at the center of the NP, while the Pt trace has two side peaks. In addition, Figure 5e shows the Au, Pt, and Au+Pt overlay EDS elemental mappings results clearly showed that Au is present in the core and Pt is localized in the shell, further suggesting that the formation core–shell structure instead of nanoalloy.

### 3.2. Electrocatalytic Measurements

**Figure 6:**
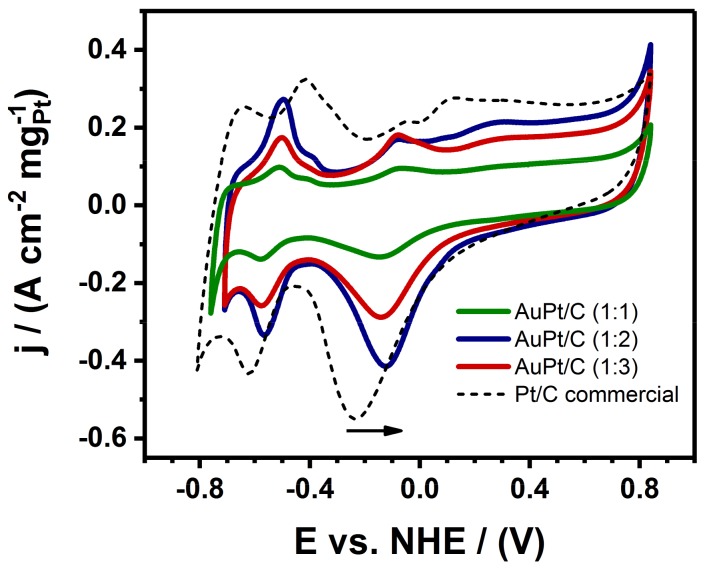
Voltammograms (CVs) of carbon supported Au@Pt/C NPs catalysts with different nominal compositions and Pt/C commercial in a 0.3 M aqueous solution of KOH at a scan rate of 50 mVs^−1^.

Figure 6 displays the cyclic voltammetry (CV) curves of the three different samples of Au@Pt NPs with varying ratios and supported on Vulcan carbon as well as the commercial Pt/C for comparison all of them in a 0.3 M aqueous KOH solution, using a potential ranging from −0.8 to 0.8 eV (vs. NHE) at a scan rate of 50 mVs^−1^. All the three studied catalysts showed distinct charge–discharge characteristics in the potential peaks from −0.8 to −0.4 eV (vs. NHE) as resulted from the hydrogen adsorption/desorption reactions [46]. For all Au@Pt/C NPs, the Au oxide reduction peak is not present, which is located at higher potential than Pt and it is characteristic in Au-Pt alloy samples [18], therefore the results indicate that the Au core is completely coated by Pt atoms [47]. The peak potential of reduction of Pt oxide species shifts to higher potential when Pt is covered with Au, from −0.22 to −0.12 eV. It is evident from the Figure 6, the three studied catalysts exhibit similar trend in the CV cycles, however the Au@Pt/C (1:2) catalysts showed remarkably higher reduction current was observed in comparison with both of the Au@Pt/C (1:1), and Au@Pt/C (1:3) catalysts, suggesting that the Au@Pt/C (1:2) catalyst possessed the highest metal electrochemical catalytic area.

**Figure 7:**
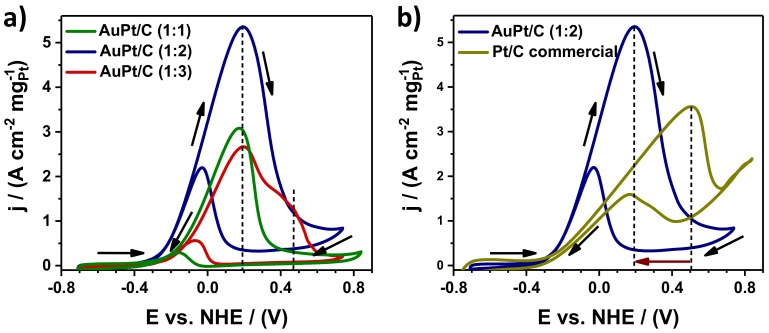
CV profiles of the catalysts for methanol oxidation reaction. CV curves for (**a**) Au@Pt/C NPs with different nominal compositions and (**b**) commercial Pt/C electrocatalysts and Au@Pt/C (1:2) NPs in 0.3 M KOH + 1 M MeOH mixed solution at a sweep rate of 20 mVs^−1^.

The electrocatalytic activities of the obtained Au@Pt/C NPs samples were studied for MOR and the results are compared. Figure 7a shows the CV curves recorded from the Au@Pt/C (1:1), Au@Pt/C (1:2), and Au@Pt/C (1:3) catalysts for MOR in a nitrogen-purged 0.3 M KOH + 1 M MeOH mixed solution at a scan rate of 20 mVs^−1^. MOR is characterized by well-separated anodic peaks in the forward and reverse scans. The forward-potential scan showed that peak current density for MOR for Au@Pt/C (1:2) was 5.69 A/cm^2^mg_Pt_, which is 2-times greater than that of the Au@Pt/C (1:3) catalysts (2.66 A/cm^2^mg_Pt_), and 1.7-times higher than that of the Au@Pt/C (1:1) catalyst (3.08 A/cm^2^mg_Pt_). From the CV results it is evident that the Au@Pt/C (1:2) catalyst exhibited significantly enhanced electrocatalytic activity toward methanol electro-oxidation. In the forward scan, all the samples show an anodic peak (*I_f_*) observed at approximately 0.19 eV vs NHE, which could be associated with the characteristic methanol oxidation on the electrode surface. In addition to the peak derived from methanol oxidation, there is also a characteristic peak in the reverse scan (*I_b_*), which is attributed to the removal of CO-like intermediate species during MOR. The ratio between the anodic and cathodic peaks current is used to evaluate the anti-poisoning ability of a catalyst [48,49]. The ratios (*I_f_/I_b_*) of the three catalysts show the next descending order: AuPt/C (1:2) >AuPt/C (1:1) >AuPt/C (1:3).

The sample with higher Pt content (Au@Pt/C (1:3)) shows an extra anodic peak at approximately 0.47 eV vs NHE, which could be attributed to methanol oxidation on Pt site only due to this potential is observed also to the anodic peak in the forward-potential for MOR for commercial Pt/C catalyst and is in agreement with the reaction mechanism for MOR, this is, the reaction must involve the either the adsorption then dissociation of an oxygen donor that is a hydroxyl in alkaline media. The state-of-the-art Pt catalyst can only do this process above 0.4–0.5 eV vs. NHE.

In order to confirm the higher electrocatalytic activity of Au@Pt/C catalyst, we carried out the electrocatalytic activity on commercial Pt/C catalysts under the same condition and the results were compared with the Au@Pt/C (1:2) catalyst. Figure 7b depicts the CV curves of Au@Pt/C (1:2) and Pt/C catalysts for MOR, which revealed that the Au@Pt/C (1:2) catalyst exhibited remarkably higher anodic current density (5.69 A/cm^2^mg_Pt_), which is almost 1.5-fold greater than that of the commercial Pt/C catalyst (3.56 A/cm^2^mg_Pt_). Pt/C catalyst shows an anodic peak at approximately 0.5 eV vs NHE, above than that of Au@Pt/C (1:2) catalyst (0.19 eV vs NHE). The negative shift of the anodic peak is associated with the loading amount of Pt on the Au@Pt NPs and direct methanol oxidation. The ratio *I_f_/I_b_* of Au@Pt/C (1:2) catalyst is greater than of the ratio *I_f_/I_b_* of Pt/C-commercial catalyst, which confirms the improved CO-tolerance capability of AuPt/C (1:2). These results agree with the reported using density functional theory calculations to determine the stability of Pt-Au clusters in the presence of CO. The results showed that in presence of CO, Pt diffuses to the surface, allowing the formation of strong Pt-CO bonds [50]. In contrast, when Pt metal is encapsulated by Au (Pt@Au), the catalytic activity diminished due to the CO oxidation does not occur at the cluster surfaces that are initially pure Au [51].

## 4. Conclusions

In summary, an easy and efficient synthetic strategy to the controlled synthesis of Au@Pt core–shell NPs with Pt-enriched surface as an efficient electrocatalysts for methanol electro-oxidation has been reported. By varying the ratio between Au/Pt, the elemental distribution and the Pt-coverage onto AuNPs can be easily controlled. Detailed structural characterization using HAADF-STEM analysis revealed that the effective formation of Pt-monolayers onto the AuNPs surface. At an optimized Pt ratio in the Au@Pt/C (1:2) NPs showed greatly enhanced electrocatalytic activity toward methanol oxidation under alkaline condition in comparison with the other obtained Au@Pt/C NPs as well as Pt/C commercial catalysts. The results confirmed that the Pt-monolayer enriched surface in the Au@Pt core–shell NPs increases the electrocatalytic activity for methanol oxidation. Our present synthetic strategy for preparing Au@Pt core–shell NPs with superior electrocatalytic activity optimization can be promising for their use in various catalytic reactions.
